# Pulsed Thulium:Yag, Thulium Fiber, and Holmium:Yag Lasers in Mini-PCNL: A Three-Arm Comparative Cohort Study

**DOI:** 10.3390/jcm15187049

**Published:** 2026-09-11

**Authors:** Silvia Proietti, Cristian Axel Hernandez Gaytan, Jorge Augusto Alcacio-Mendoza, Franco Gaboardi, Guido Giusti

**Affiliations:** 1 Department of Urology, IRCCS San Raffaele Hospital, 20132 Milan, Italy; 2 Department of Urology, Instituto Nacional de Ciencias Médicas y Nutrición “Salvador Zubirán”, Mexico City 14080, Mexico

**Keywords:** urolithiasis, mini-PCNL, pulsed thulium:YAG, holmium:YAG laser, thulium fiber laser, endourology

## Abstract

**Background & Objectives:** Laser lithotripsy is the standard energy source for mini-percutaneous nephrolithotomy (mini-PCNL). Although Holmium:YAG (Ho:YAG), Thulium Fiber Laser (TFL), and pulsed Thulium:YAG (p-Tm:YAG) are currently available for clinical use, direct comparisons among all three laser technologies are lacking. This study aimed to compare the efficacy, safety, and procedural performance of Ho:YAG, TFL, and p-Tm:YAG during mini-PCNL. The primary endpoint was the stone-free rate (SFR). Secondary outcomes included complications, operative time, lasing time, total energy delivered, and laser-related procedural metrics. **Methods:** Consecutive patients treated with p-Tm:YAG during mini-PCNL for renal stones were prospectively enrolled and compared with two cohorts from our institutional database treated with Ho:YAG or TFL. All procedures were performed using the same mini-PCNL technique and predefined laser settings selected to achieve the closest technically feasible match among the three platforms. **Results:** Ninety patients were included (30 per group). No significant differences in SFR were observed among Ho:YAG, TFL, and p-Tm:YAG (*p* = 0.93), or in overall complication rates (*p* = 0.93). After adjusting for stone volume, density, composition, and location, the laser platform was not independently associated with SFR, whereas stone volume was the only independent predictor of stone-free status. TFL exhibited a significantly longer operative time than Ho:YAG and a longer lasing time than both Ho:YAG and p-Tm:YAG, and was associated with greater total energy delivery, lower ablation efficiency, and lower ablation speed. Conversely, p-Tm:YAG demonstrated a significantly shorter lasing time than TFL and an ablation efficiency similar to that of Ho:YAG, with lasing time and ablation speed values between those observed with Ho:YAG and TFL under the evaluated settings. Laser efficacy did not differ significantly among the three groups. **Conclusions:** No significant differences in stone-free or safety outcomes were observed among the three laser platforms in mini-PCNL. Although significant differences were observed in lasing time, ablation speed, and energy utilization, p-Tm:YAG demonstrated an intermediate procedural profile under the operating parameters evaluated in this study. Overall, the present findings suggest that p-Tm:YAG represents a feasible treatment option for mini-PCNL, although larger randomized studies are needed before firm conclusions regarding relative platform performance can be drawn.

## 1. Introduction

Miniaturized percutaneous nephrolithotomy (Mini-PCNL) has become an established treatment option for renal calculi, combining high stone-free rates with reduced morbidity compared with standard PCNL [1,2]. The development of dedicated miniaturized instruments, together with continuous advancements in laser technology, has contributed to its widespread adoption in contemporary endourological practice.

Laser lithotripsy currently represents the main energy source for stone treatment during Mini-PCNL [3]. Over the last decade, different laser platforms have become available, including the Holmium:YAG (Ho:YAG) laser, the Thulium Fiber Laser (TFL), and, more recently, the pulsed Thulium:YAG (p-Tm:YAG) laser [4,5,6,7]. These systems differ substantially in terms of wavelength, water absorption, pulse architecture, peak power, and laser–stone interaction mechanisms, potentially influencing lithotripsy efficiency and procedural performance.

While several studies have evaluated individual laser platforms and a limited number of pairwise comparisons have been reported [8,9,10,11], a direct comparison of all three currently available laser technologies in the Mini-PCNL setting is lacking. Given the reduced tract size and the technical constraints inherent to miniaturized percutaneous surgery, parameters such as lasing time, operative time, energy delivery, stone-free rate (SFR) and complications may be particularly relevant when assessing the clinical performance of different laser systems.

Therefore, the aim of the present study was to compare the efficacy and perioperative and postoperative outcomes of Ho:YAG, TFL, and p-Tm:YAG lasers in patients undergoing Mini-PCNL.

## 2. Methods

Consecutive adult patients (≥18 years) undergoing mini-percutaneous nephrolithotomy (mini-PCNL) for renal stones with the p-Tm:YAG laser (Dornier Thulio^®^; Dornier MedTech Systems GmbH, Wessling, Germany) were prospectively enrolled. These patients were compared with two cohorts from our retrospectively collected institutional database, including patients treated with either a 150-W Ho:YAG laser (Quanta System S.p.A, Samarate, Italy) or TFL (Fiber Dust Pro^®^; Quanta System S.p.A, Samarate, Italy). All patients across the three groups were treated during the same calendar year at the same high-volume referral center. All procedures were performed by two experienced endourologists using the same standardized mini-PCNL technique and instrumentation in all three groups. Patients with incomplete clinical or perioperative data were excluded from the analysis. The study was conducted in accordance with the Declaration of Helsinki and approved by the local Ethics Committee. Informed consent was obtained from all participants.

Routine preoperative and 1-month postoperative evaluation included medical history, physical examination, urinalysis, urine culture, and blood tests. Non-contrast computed tomography (NCCT) was performed in all patients both preoperatively and 1 month after surgery. Stone burden was calculated as the cumulative stone volume using the ellipsoid formula (mm^3^). Patients were considered stone-free if no residual fragments were detected on postoperative NCCT, defined as SFR level 0 in Somani’s classification [12].

Operative time was defined as the interval from insertion of the first endoscope to completion of ureteral stent placement. Perioperative and postoperative complications were graded according to the Clavien-Dindo classification [13]. Mean Hounsfield unit (HU) density was recorded as a surrogate marker of stone hardness.

Baseline patient characteristics, including age, sex, body mass index (BMI), and stone laterality, were recorded. Stone location was categorized into three standardized groups: single calyx (upper, middle, or lower calyx), renal pelvis, or multiple locations (defined as stones involving two or more anatomical sites). Stone composition was categorized into two groups: calcium oxalate monohydrate (COM) stones, including pure COM stones and mixed stones in which COM was the predominant component, and non-COM stones.

All procedures were performed using the previously described technique [14] and the MIP-M set (Karl Storz, Tuttlingen, Germany). A 550-μm laser fiber was used. Laser settings were 1.5 J and 30 Hz in short-pulse (SP) mode for the Ho:YAG and TFL groups, whereas the p-Tm:YAG group was treated using 1.5 J and 25 Hz in SP mode. The selected settings were chosen to achieve the closest technically feasible match among the available laser systems. Identical frequency settings could not be applied because the p-Tm:YAG platform was limited to 25 Hz under the selected operating conditions.

Operative variables included total operative time (min), lasing time (min) and total energy delivered (J).

### 2.1. Outcome Measures

The primary endpoint was the SFR, assessed by NCCT performed 1 month after surgery. Secondary endpoints included overall postoperative complications (Clavien-Dindo grade ≥ 1), total operative time (min), lasing time (min), total energy delivered (J), laser ablation efficiency (mm^3^/J), laser ablation speed (mm^3^/s), laser efficacy (mm^3^/min), and laser energy consumption (J/mm^3^). Stone volume used for the calculation of laser-related metrics was derived from preoperative NCCT. According to the standardized terminology proposed by Kwok et al. [15], laser ablation efficiency was defined as stone volume divided by total laser energy, laser ablation speed as stone volume divided by lasing time, laser efficacy as stone volume divided by total operative time, and laser energy consumption as total laser energy divided by stone volume. Total laser energy was recorded directly from the laser system for each procedure. All derived laser-related metrics were calculated at the individual patient level before group-level summary statistics were obtained.

### 2.2. Statistical Analysis

Continuous variables were tested for normality using the Shapiro–Wilk test. Because the continuous variables of interest, including stone volume, operative time, lasing time, and total energy delivered, showed a non-normal distribution, non-parametric methods were applied for unadjusted comparisons. Continuous variables are presented as median [interquartile range (IQR)], whereas categorical variables are reported as frequencies and percentages. The study was neither designed nor powered to assess equivalence or non-inferiority; accordingly, equivalence among the three laser platforms cannot be established from the present findings.

Comparisons among the three laser groups were performed using the Kruskal–Wallis test for continuous variables and Pearson’s chi-square test or Fisher’s exact test, as appropriate, for categorical variables. When the overall Kruskal–Wallis test yielded a *p*-value < 0.05, post hoc pairwise comparisons were performed using the Mann–Whitney U test with Holm correction for multiple testing. Effect sizes for pairwise comparisons were calculated as r = |Z|/√N.

To assess whether laser modality was independently associated with the study outcomes after adjustment for potential confounding factors, multivariable regression analyses were performed. Binary logistic regression was used to identify independent predictors of stone-free status (SFR), with SFR entered as the dependent variable and laser platform as the main independent variable, while stone volume, mean Hounsfield Unit (HU) density, stone composition, and stone location were simultaneously included in the model as covariates (stone location was entered as two dummy variables, with single calyceal stones as the reference category). Ordinary least squares (OLS) linear regression models were used to evaluate continuous outcomes, including operative time, lasing time, and total energy delivered, using the same covariates.

Model assumptions were assessed by checking residuals for normality with the Shapiro–Wilk test and evaluating multicollinearity via the condition number. A sensitivity analysis restricted to the COM-predominant subgroup (n = 53) was performed to evaluate the consistency of the findings within a more homogeneous stone composition subgroup.

All statistical tests were two-sided, and a *p*-value < 0.05 was considered statistically significant unless otherwise specified. Statistical analyses were performed using Python version 3.12 (SciPy version 1.14 and statsmodels version 0.14).

## 3. Results

Ninety patients (47 males, 43 females) were included, with 30 patients in each laser group. Table 1 summarizes baseline demographics and stone characteristics. No statistically significant differences were observed among the three groups in age, BMI, stone volume, mean HU density, gender distribution, stone composition, or stone location (Table 1).

No significant differences in SFR were observed among the three modalities: 83.3% (25/30) for Ho:YAG, 80.0% (24/30) for TFL, and 80.0% (24/30) for p-Tm:YAG (*p* = 0.93).

In the multivariable logistic regression analysis adjusting for stone volume, mean HU density, composition, and location, SFR did not significantly differ among the three laser groups, with neither TFL (OR = 0.76, 95% CI: 0.15–3.79, *p* = 0.74) nor p-Tm:YAG (OR = 0.76, 95% CI: 0.15–3.80, *p* = 0.73) differing significantly from Ho:YAG laser. The only independent predictor of SFR was stone volume (OR = 0.9992 per mm^3^, 95% CI: 0.9987–0.9998, *p* = 0.007).

No significant differences in complication rates were observed among the three groups: 16.7% (5/30) for Ho:YAG, 20.0% (6/30) for TFL, and 20.0% (6/30) for p-Tm:YAG (*p* = 0.93). Most complications were Clavien–Dindo grade II (13/17, 76.5%), with only two grade III events observed in the entire cohort, one each in the Ho:YAG and TFL groups. No grade IV or V complications occurred.

Operative time differed significantly among the three groups (*p* = 0.014). Post hoc analysis showed a significantly longer operative time with TFL than with Ho:YAG (*p* = 0.011), whereas no significant differences were observed between p-Tm:YAG and either Ho:YAG or TFL.

Lasing time also differed significantly among the three groups (*p* < 0.001), with the longest time observed with TFL (8.0 min [IQR 5.5–13.4]), followed by p-Tm:YAG (5.5 min [IQR 2.8–6.4]) and Ho:YAG (2.9 min [IQR 1.5–5.4]), with significant differences across all pairwise comparisons.

Similarly, total energy delivery differed significantly among the three groups (*p* = 0.001). TFL required significantly more energy than Ho:YAG (13,214 vs. 6836 J; *p* < 0.001), whereas no significant differences were observed between TFL and p-Tm:YAG or between p-Tm:YAG and Ho:YAG after Holm correction.

**Figure 1:**
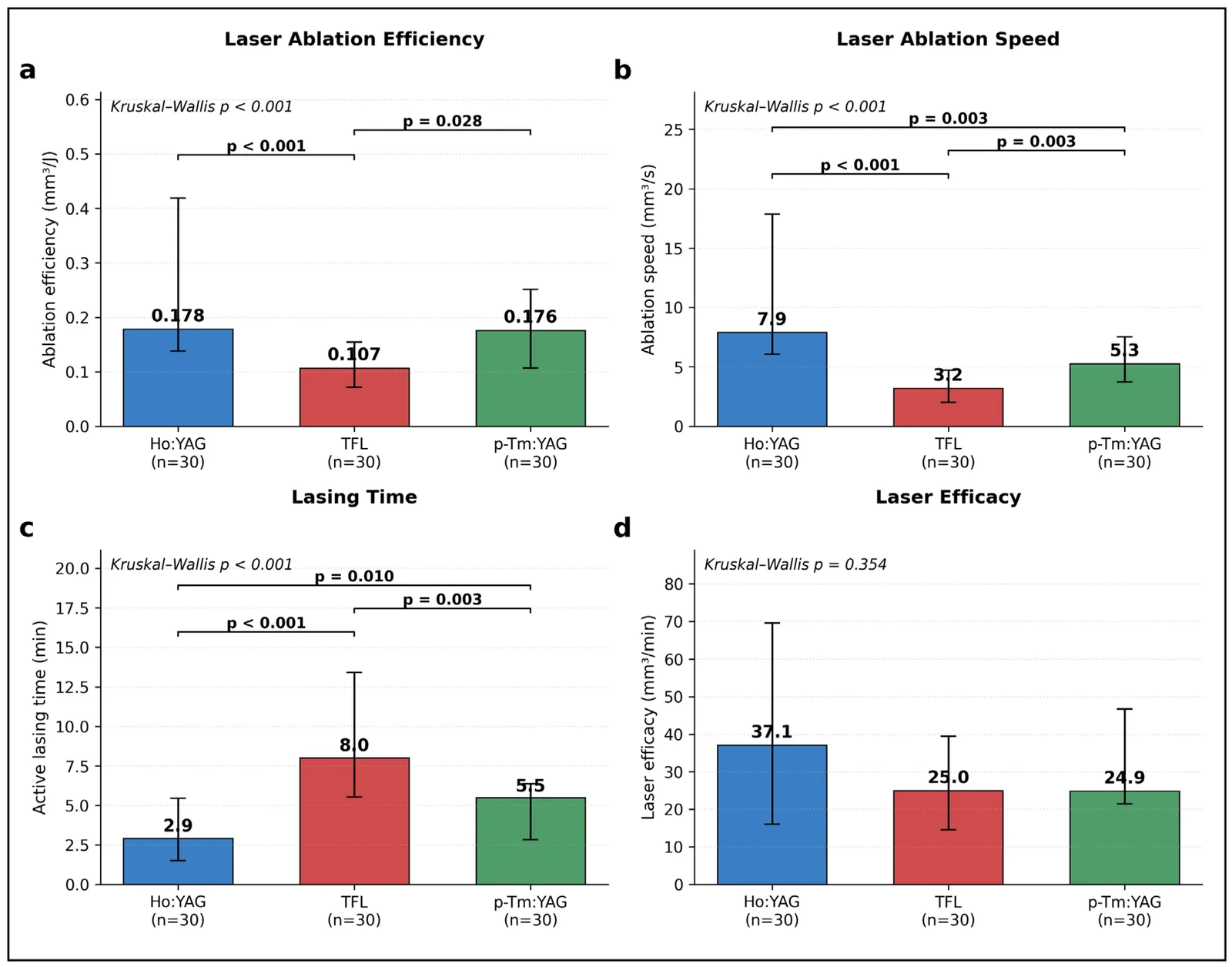
Comparison of laser performance metrics among Ho:YAG, TFL, and p-Tm:YAG. (**a**) Laser ablation efficiency (mm^3^/J), (**b**) laser ablation speed (mm^3^/s), (**c**) lasing time (min), and (**d**) laser efficacy (mm^3^/min).

Analysis of the predefined laser-related metrics further highlighted these differences. TFL showed a significantly lower ablation efficiency (0.107 mm^3^/J) than both Ho:YAG (0.178 mm^3^/J) and p-Tm:YAG (0.176 mm^3^/J). Likewise, ablation speed varied significantly among the three groups (*p* < 0.001), with values of 7.91 mm^3^/s for Ho:YAG, 5.26 mm^3^/s for p-Tm:YAG, and 3.19 mm^3^/s for TFL. In contrast, laser efficacy did not differ significantly among the three technologies (*p* = 0.35) (Figure 1, Table 2).

**Figure 2:**
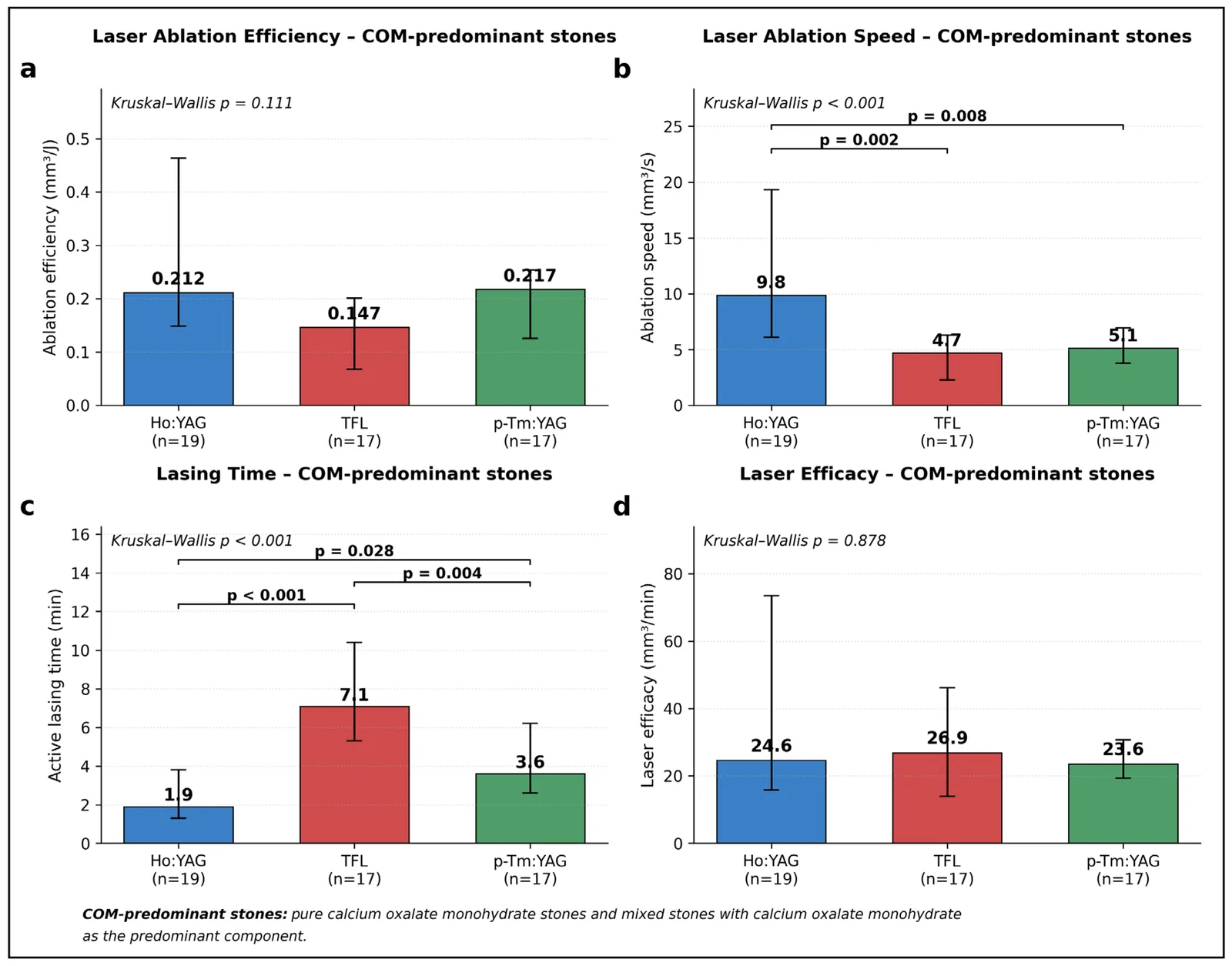
Comparison of laser performance metrics among Ho:YAG, TFL, and p-Tm:YAG in the COM-predominant stone subgroup (n = 53). (**a**) Laser ablation efficiency (mm^3^/J), (**b**) laser ablation speed (mm^3^/s), (**c**) lasing time (min), and (**d**) laser efficacy (mm^3^/min).

No significant differences in SFR were noted in the COM-predominant subgroup (84.2%, 82.4%, and 88.2% for Ho:YAG, TFL, and p-Tm:YAG, respectively). Time-based metrics mirrored the findings of the overall cohort: TFL required the longest lasing time, which was significantly longer than that of both Ho:YAG (*p* < 0.001) and p-Tm:YAG (*p* = 0.004), and exhibited a slower ablation speed than Ho:YAG (*p* = 0.002). Ablation efficiency did not differ significantly among the three lasers (*p* = 0.11), and laser efficacy also did not differ significantly among the groups (*p* = 0.88) (Figure 2).

## 4. Discussion

To our knowledge, this is the first study to directly compare the three laser platforms currently available for lithotripsy in mini-PCNL: the Ho:YAG laser, the thulium fiber laser (TFL), and the p-Tm:YAG laser. No significant differences in stone-free or complication rates were observed among the three groups; however, significant differences in laser-related procedural parameters were identified among the three technologies. TFL showed the longest lasing time, which was significantly longer than that of both Ho:YAG and p-Tm:YAG, whereas the lasing time for p-Tm:YAG was intermediate between those observed with Ho:YAG and TFL. Operative time was significantly longer with TFL than with Ho:YAG, while p-Tm:YAG did not differ significantly from either platform. Overall, these findings indicate differences in procedural performance among the three laser platforms with the specific devices and operating parameters evaluated in this study.

These findings should be interpreted in light of the well-established differences in wavelength, water absorption, pulse architecture, and laser–stone interaction among the three platforms [5,16,17]. TFL emits at a wavelength of approximately 1940 nm and is characterized by markedly higher water absorption and a shallower optical penetration depth than Ho:YAG [5,17]. These optical properties, together with differences in pulse architecture and peak power, may influence the balance between photothermal and photomechanical effects during laser lithotripsy [16]. Although both TFL and p-Tm:YAG operate at wavelengths characterized by high water absorption, the distinct pulse architecture and peak power profile of p-Tm:YAG differentiate it from TFL and may result in a different laser–stone interaction [18,19]. These variations in optical properties and energy delivery may, at least in part, help explain the differences in procedural parameters observed among the three platforms. Of note, p-Tm:YAG was operated at 25 Hz because of the technical specifications of the system, whereas Ho:YAG and TFL were used at 30 Hz. Consequently, the nominal average power was lower for p-Tm:YAG (37.5 W) than for Ho:YAG and TFL (45 W). Therefore, the present findings should be interpreted as reflecting the combined effect of laser technology and the specific treatment settings employed. Because frequency contributes directly to power delivery and total energy deposition, the relative influence of the laser platform versus treatment parameters cannot be fully disentangled in the present study. Accordingly, the observed differences should not be regarded as inherent performance characteristics of the respective laser technologies.

The analysis of standardized laser-related metrics, calculated using CT-derived stone volume measurements, provided further insight into the observed procedural differences. TFL demonstrated lower ablation efficiency than both Ho:YAG and p-Tm:YAG. Ablation speed was highest with Ho:YAG, lowest with TFL, and intermediate with p-Tm:YAG, with significant differences noted among the three platforms. Conversely, laser efficacy, expressed as stone volume treated per minute of total operative time, did not differ significantly among the three technologies. These metrics were calculated according to the standardized definitions proposed by Kwok et al. [15]; however, in the present clinical setting, they should be interpreted as comparative measures of laser-related procedural performance rather than direct measurements of the intrinsic physical ablation capability of the laser source. In particular, laser ablation speed was not normalized for average power, in accordance with the standardized definition, and the lower nominal average power used with p-Tm:YAG should therefore be considered when interpreting this parameter. Differences in these laser-related metrics may become less pronounced when assessed within the overall surgical procedure, where fragment evacuation, endoscopic manipulation, visibility, irrigation dynamics, and other non-lasing steps also contribute substantially to operative time.

The three study groups were comparable with respect to demographic variables and stone characteristics, including stone size, volume, density, composition, and multiplicity. Therefore, the observed differences in laser-related procedural parameters are unlikely to be driven solely by major baseline heterogeneity but should instead be interpreted as reflecting the combined influence of the laser platforms and the specific operating parameters evaluated in this study.

Interestingly, in the COM subgroup, TFL showed a longer lasing time than both Ho:YAG and p-Tm:YAG, whereas comparable values were observed between Ho:YAG and p-Tm:YAG. This finding appears to contrast with the in vitro study by Panthier et al., which demonstrated higher ablation rates with TFL for COM stones [20]. However, this apparent discrepancy may reflect the fundamental differences between experimental and clinical settings. While in vitro ablation rates assess laser–stone interaction under controlled experimental conditions, clinical lasing time reflects a more complex and dynamic process that is influenced by several procedural factors. Therefore, superior ablation performance under laboratory conditions may not necessarily translate into a shorter lasing time in clinical practice. Nevertheless, given the exploratory nature and limited sample size of this subgroup analysis, these findings should be interpreted cautiously.

Our results align with those recently reported by Vergamini et al., who observed shorter operative time and greater intraoperative laser efficiency with Ho:YAG compared with TFL during mini-PCNL [8]. Similarly, lower ablation efficiency and ablation speed were observed with TFL in our cohort. However, these differences should be interpreted in the context of the specific devices and operating parameters evaluated in each study and cannot be attributed solely to intrinsic differences between the laser technologies.

Our findings differ, however, from those of a previous comparative study evaluating Ho:YAG and p-Tm:YAG, which reported significantly shorter lasing and operative times, as well as higher stone-free rates, with p-Tm:YAG [9]. In our cohort, p-Tm:YAG exhibited a longer lasing time than Ho:YAG, whereas no significant difference in overall operative time or stone-free rate was observed between the two technologies. These discrepancies may, at least in part, be explained by differences in baseline stone characteristics between the study populations. Notably, the p-Tm:YAG group in the aforementioned study included a significantly higher proportion of patients with a single stone, whereas stone multiplicity was similarly distributed among the three study groups in our cohort. Given the potential impact of stone burden and complexity on procedural efficiency, this imbalance may have contributed to the differences between the two studies. Furthermore, direct comparisons between the two studies are inherently limited, as a different p-Tm:YAG platform was evaluated, laser settings were adjusted intraoperatively according to stone composition, and postoperative stone-free status was assessed using a non-uniform imaging protocol rather than systematic NCCT in all patients.

Moreover, a prospective randomized trial comparing TFL and Ho:YAG during mini-PCNL demonstrated a significant advantage of TFL in terms of operative efficiency [21]. By contrast, in our study, TFL was associated with significantly longer lasing and operative times than Ho:YAG. These findings differ from those reported by Mahajan et al., who demonstrated superior procedural efficiency with TFL during mini-PCNL. Nevertheless, the two studies were conducted using different treatment protocols. While laser settings in our study were standardized across all procedures, Mahajan et al. allowed adjustments within a predefined range during surgery. This methodological difference may have influenced procedural efficiency and should be considered when interpreting these discrepant results.

Recently, Perri et al. compared Magneto pulse-modulated Ho:YAG and TFL during mini-PCNL, reporting comparable stone-free and complication rates, with no significant differences in operative or lasing times, despite a trend toward greater dusting efficiency with Magneto technology [10]. Unlike their study, where laser settings were tailored according to stone characteristics, our analysis was performed using predefined settings for each laser platform, thereby minimizing a potential source of intraoperative variability and allowing a more direct comparison of the procedural metrics observed under the evaluated settings. Furthermore, by including p-Tm:YAG alongside Ho:YAG and TFL, our study expands the available clinical evidence, providing the first clinical comparison of all three platforms in the mini-PCNL setting.

Interestingly, our findings are in line with those of a recent comparative study evaluating Ho:YAG and p-Tm:YAG in mini-PCNL, which also reported no significant differences in clinical outcomes despite differences in laser-related procedural parameters [11]. By extending the comparison to include TFL, our study provides additional clinical data on the procedural characteristics of p-Tm:YAG relative to both Ho:YAG and TFL under the specific operating parameters evaluated. No significant differences in stone-free or complication rates were observed among the three groups.

Our study did not demonstrate any significant differences in SFR among the three laser platforms. Moreover, after adjusting for stone volume, density, composition, and location, laser technology was not independently associated with SFR, whereas stone volume emerged as the only independent predictor of stone-free outcome. These findings suggest that, within the present cohort, stone burden may play a greater role in determining final stone clearance than the specific laser source used. Nevertheless, given the limited sample size, the absence of statistically significant differences in SFR should not be interpreted as evidence of clinical equivalence among the three laser platforms. The choice of laser technology may therefore have a greater impact on procedural efficiency and energy utilization than on the likelihood of achieving stone-free status. However, the significant differences in lasing time and laser efficiency parameters should not be overlooked. Although these differences were not reflected in stone-free outcomes, procedural efficiency remains a clinically relevant consideration, as prolonged operative and lasing times may affect operating room utilization, anaesthesia exposure, and overall procedural burden. Therefore, despite the absence of significant differences in stone-free rates, the three laser platforms showed distinct procedural and energy-related metrics under the operating parameters evaluated in this study. Accordingly, similar stone-free rates should not be interpreted as demonstrating either clinical equivalence or equivalent laser-related procedural performance.

All procedures in the present series were performed using the MIP-M mini-PCNL instrument. Stone fragment evacuation was achieved through the vacuum-cleaner effect, without the use of dedicated active suction-assisted sheaths. More recently, technological advances in percutaneous access systems have led to the development of access sheaths incorporating active suction mechanisms, aimed at improving fragment clearance and procedural efficiency. Several investigations have suggested that these devices may facilitate stone fragment evacuation and contribute to shorter operative times during mini-PCNL [22].

Data regarding the interaction between different laser technologies and percutaneous suction-assisted systems during mini-PCNL remain limited. A comparative study evaluating TFL and Ho:YAG in a suction-assisted mini-PCNL setting reported no significant differences in stone-free rates between the two laser platforms [23]. Similarly, a study comparing low-power and high-power Ho:YAG lasers failed to demonstrate any significant advantage in terms of stone clearance [24]. These findings are consistent with our results and suggest that final stone-free outcomes may be largely independent of the laser source employed. Nevertheless, stone-free status alone may not fully capture procedural performance. Given the significant differences in lasing time, energy utilization, and ablation-related metrics observed in our study, future investigations comparing Ho:YAG, TFL, and p-Tm:YAG in combination with active suction systems are warranted. Such studies may clarify whether optimized fragment evacuation can attenuate or amplify differences in laser-related procedural efficiency among these technologies.

The present study has several limitations. First, the non-randomized design and relatively limited sample size may have introduced selection bias and residual confounding. In particular, the p-Tm:YAG group was prospectively collected and compared with Ho:YAG and TFL groups derived from a previously established institutional cohort. Although the three groups were comparable in terms of demographic and stone characteristics, unmeasured differences related to patient selection and other potential confounding factors cannot be completely excluded. To mitigate these potential sources of bias, all patients across the three groups were treated during the same calendar year at the same high-volume referral center, and all procedures were performed by two experienced endourologists using the same standardized mini-PCNL technique and instrumentation. The contemporaneous inclusion of the three cohorts and the consistency of the surgical setting and technique may have reduced, but cannot eliminate, the potential influence of temporal and procedural variability. Despite these elements of standardization, the mixed prospective-retrospective design may still have introduced unmeasured variations in patient selection, surgeon-related factors, and perioperative management that could not be fully accounted for by multivariable adjustment.

Furthermore, the relatively small sample size (30 patients per group) limited the statistical power to detect modest but potentially clinically relevant differences in SFR and complication rates. The study was neither designed nor powered to assess equivalence or non-inferiority; accordingly, equivalence among the three laser platforms cannot be established from the present findings.

Second, although laser settings were selected to be as comparable as technically feasible, identical frequency settings could not be used across all three platforms, as the p-Tm:YAG system did not allow a 30-Hz setting and was therefore operated at 25 Hz. Consequently, p-Tm:YAG was utilized at a lower nominal average power (37.5 W) than Ho:YAG and TFL (45 W). Because repetition frequency and average power may influence energy delivery, lasing time, and derived laser-related metrics, the relative contribution of the laser platform and treatment parameters cannot be fully disentangled in the present study. Accordingly, the observed differences should be interpreted in the context of the specific devices and operating parameters evaluated rather than as inherent performance characteristics of the respective laser technologies.

Third, although ablation efficiency, ablation speed, energy consumption, and laser efficacy were calculated using objective CT-derived stone volume measurements and standardized definitions applied uniformly across all groups, these parameters remain clinical laser-related metrics rather than direct measurements of the intrinsic ablation capability of the laser source. They reflect the combined effect of laser–stone interaction and procedural factors such as fragment evacuation, endoscope handling, visibility, and irrigation dynamics. Furthermore, these metrics were derived from preoperative stone volume rather than direct measurement of the actual stone volume ablated. Therefore, they should be interpreted as comparative measures of laser-related procedural performance rather than intrinsic properties of the laser source.

Finally, the subgroup analysis according to stone composition, particularly for COM stones, was exploratory and limited by the small number of patients within individual composition subgroups. These findings should therefore be interpreted cautiously and require confirmation in adequately powered prospective studies.

Despite these limitations, this study provides the first direct three-way clinical comparison of Ho:YAG, TFL, and p-Tm:YAG in mini-PCNL. Our findings showed no significant differences in stone-free or complication rates among the three groups, while significant variations were observed in time- and energy-related procedural metrics. With the specific devices and operating parameters evaluated in this study, p-Tm:YAG demonstrated an intermediate procedural profile between Ho:YAG and TFL, particularly in terms of lasing time and ablation speed, although its ablation efficiency was more similar to that of Ho:YAG than to that of TFL. This intermediate profile should be interpreted as an observation under the specific conditions evaluated rather than as an inherent characteristic of p-Tm:YAG technology. Prospective randomized studies with larger sample sizes and appropriately standardized operating parameters are warranted to confirm these findings and further clarify the role of each laser technology in mini-PCNL.

## 5. Conclusions

The three laser platforms showed no significant differences in stone-free or safety outcomes in mini-PCNL. Although significant differences were observed in lasing time, ablation speed, and energy utilization, p-Tm:YAG demonstrated an intermediate procedural profile under the operating parameters evaluated in this study, exhibiting time-related outcomes between Ho:YAG and TFL and energy-efficiency measures similar to those of Ho:YAG. These results should be interpreted in the context of the specific devices, operating parameters, and study design evaluated. Overall, the present findings suggest that p-Tm:YAG represents a feasible treatment option for mini-PCNL, although larger randomized studies are needed before firm conclusions regarding relative platform performance can be drawn.

## Figures and Tables

**Table 1:** Baseline characteristics by laser modality.

Variable	Ho:YAG (n = 30)	TFL (n = 30)	p-Tm:YAG (n = 30)	*p*-Value
Age (years)	54 (48–62)	59 (46–63)	61 (48–66)	0.43 ^a^
BMI (kg/m^2^)	27 (24–28)	25 (23–28)	24 (22–27)	0.26 ^a^
Male sex, n (%)	17 (56.7)	14 (46.7)	16 (53.3)	0.73 ^b^
Stone volume (mm^3^)	1549 (813–2202)	1361 (720–2533)	1298 (965–2547)	0.95 ^a^
HU density	1144 (906–1362)	1220 (1000–1394)	1211 (943–1342)	0.54 ^a^
Stone composition				0.83 ^b^
COM-stones	19(63.3)	17 (56.7)	17 (56.7)	
Non-COM stones	11 (36.7)	13 (43.3)	13 (43.3)	
Stone location				0.85 ^b^
Single calyceal	16 (53.3)	14 (46.7)	12 (40.0)	
Renal pelvis	8 (26.7)	10 (33.3)	12 (40.0)	
Multiple sites	6 (20.0)	6 (20.0)	6 (20.0)	

^a^ Kruskal–Wallis test; ^b^ Pearson’s chi-square test; Data are presented as median (IQR), unless otherwise specified.

**Table 2:** Operative and clinical outcomes by laser modality.

Outcome	Ho:YAG	TFL	P-Tm:YAG	*p*-Value *
SFR, n (%)	25 (83.3)	24 (80.0)	24 (80.0)	0.93
Procedure time (min)	42.4 (34.9–58.6)	56.6 (46.6–67.4)	54.0 (42.0–64.5)	0.014
Lasing time (min)	2.9 (1.5–5.4)	8.0 (5.5–13.4)	5.5 (2.8–6.4)	<0.001
Total energy (J)	6837 (3948–11,876)	13,215 (9066–23,875)	9821 (5010–13,776)	0.001
Laser Ablation efficiency (mm^3^/J)	0.178 (0.138–0.419)	0.107 (0.072–0.155)	0.176 (0.107–0.251)	0.0007
Laser Energy consumption (J/mm^3^)	5.64 (2.50–7.24)	9.39 (6.47–13.99)	5.70 (3.99–9.39)	0.0007
Laser Ablation speed (mm^3^/s)	7.9 (6.0–17.9)	3.2 (2.0–4.7)	5.3 (3.7–7.5)	<0.0001
Laser efficacy (mm^3^/min)	37.1 (16.0–69.6)	25.0 (14.5–39.5)	24.9 (21.4–46.7)	0.35
Complications, n (%)	5 (16.7)	6 (20.0)	6 (20.0)	0.93

* Overall *p* value calculated using the Kruskal–Wallis test. Data are presented as median (IQR), unless otherwise specified. Derived laser-related metrics were calculated at the individual patient level.

## Data Availability

Proietti S has full access to all the data in the study and takes responsibility for the integrity of the data and the accuracy of the data analysis.

## Abbreviations

The following abbreviations are used in this manuscript:

Mini-PCNL	miniaturized percutaneous nephrolithotomy
Ho:YAG	Holmium:YAG
TFL	Thulium Fiber Laser
p-Tm:YAG	pulsed Thulium:YAG
SFR	stone-free rate
NCCT	Non-contrast computed tomography
COM	calcium oxalate monohydrate
HU	Hounsfield Unit
BMI	body mass index
